# Spatially Engineered WO_3_ Nanofibers on BiVO_4_: A Route to High-Efficiency Photoelectrochemical Water Splitting

**DOI:** 10.1021/acsaem.5c02686

**Published:** 2025-11-25

**Authors:** Haochen Li, Zhipeng Lin, Fei Guo, Shuhui Li, Andreas Kafizas, Christopher S. Blackman, Claire J. Carmalt

**Affiliations:** † Department of Chemistry, 4919 University College London, 20 Gordon Street, London WC1H 0AJ, U.K.; ‡ Department of Chemistry, Molecular Science Research hub, 4615 Imperial College London, London W12 0BA, U.K.

**Keywords:** WO_3_/BiVO_4_ heterojunction, aerosol-assisted chemical vapor deposition (AACVD), inverted architecture, permeable nanofibers, photoelectrochemical water splitting, charge carrier separation

## Abstract

This study employs aerosol-assisted chemical vapor deposition (AACVD) to fabricate WO_3_/BiVO_4_ heterojunction photoanodes with an inverted architecture (WO_3_ atop BiVO_4_). The unique permeable nanofiber morphology of WO_3_ provides a solution to enhance water oxidation performance. By correlating precursor volume (10–40 mL) and spatial position within the deposition chamber (inlet/mid/outlet) with film properties, we demonstrate that a midreactor position yields “grass-like” WO_3_ nanofibers (diameter: 100–230 nm, length: 3.5–3.98 μm), enabling dual functionality: (i) > 50% light transmittance to the underlying BiVO_4_ absorber, and (ii) electrolyte penetration into the heterointerface between WO_3_ and BiVO_4_. In contrast, rod-like WO_3_ produced near the inlet causes severe light scattering, reducing the incident photon-to-current efficiency (IPCE) by six times above wavelengths of 350 nm. Optimized samples, produced with a deposition volume of 30 mL to deposit WO_3_ atop of BiVO_4_ positioned in the middle of the deposition chamber (i.e., WO_3_-30/BiVO_4_-mid), achieve a photocurrent density of 0.82 mA·cm^–2^ at 1.23 V_RHE_ under 1 sun irradiance, which is 121% higher than single-layer BiVO_4_ (0.37 mA·cm^–2^) and exceeds some conventional WO_3_-under/BiVO_4_ heterojunctions in which WO_3_ is underneath BiVO_4_. Transient absorption spectroscopy confirms prolonged carrier lifetimes in our unique heterostructure through improved charge-carrier separation. This work challenges current traditional heterojunction design rules for the WO_3_/BiVO_4_ system by showcasing how permeable WO_3_ nanostructures atop BiVO_4_ photoanodes can improve light harvesting and facilitate charge-carrier separation to significantly improve activity.

## Graphical abstract

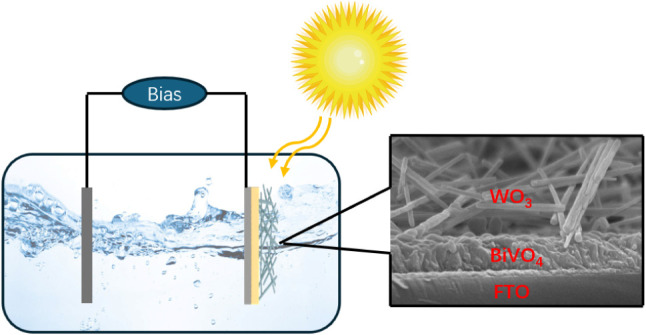

## 1 Introduction

The escalating global energy demand and the urgent need to mitigate carbon emissions have intensified research efforts toward sustainable energy technologies, particularly solar-driven hydrogen production via photoelectrochemical (PEC) water splitting.^1^ Central to this technology are semiconductor photoanodes capable of efficiently harvesting sunlight and driving the oxygen evolution reaction (OER). Among various semiconductor materials, bismuth vanadate (BiVO_4_) and tungsten trioxide (WO_3_) have garnered significant attention due to their favorable bandgap energies, chemical stability, and cost-effectiveness.^2–4^ BiVO_4_, with a suitable band energetics to facilitate OER and a bandgap of ∼2.4 eV, exhibits exceptional visible-light absorption up to 515 nm, making it a leading candidate for photoanodes. However, its practical performance is hampered by poor charge carrier mobility and severe electron–hole recombination.^5^ Conversely, WO_3_, with a wider bandgap (∼2.8 eV), offers superior electron transport properties and stability but suffers from limited visible-light utilization.^4^ These complementary properties underscore the potential of combining BiVO_4_ and WO_3_ into heterostructures to combine their respective advantages while mitigating individual drawbacks.^6^

Heterojunction engineering, particularly type-II band alignments,^7^ facilitates the spatial separation of photogenerated carriers by creating an energy cascade for charge carriers to separate in opposite directions at the materials interface. In previous reports for WO_3_/BiVO_4_ systems, the conduction band (CB) of WO_3_ lies at a more positive than that of BiVO_4_, while the valence band (VB) of BiVO_4_ is more negative than that of WO_3_.^8,9^ This alignment promotes electron transfer from BiVO_4_ to WO_3_ and hole retention in BiVO_4_, thereby enhancing charge separation and reducing recombination losses.^10^ Previous studies have demonstrated that such heterojunctions achieve higher photocurrent densities compared to their individual components, attributed to improved light harvesting and interfacial charge dynamics.^11–14^ However, the performance of these systems is highly sensitive to structural parameters, including layer thickness, crystallographic orientation, and interfacial contact quality.^15,16^ Especially for this study, since WO_3_ is on top of BiVO_4_, excessive WO_3_ growth will block light penetration into the BiVO_4_ layer, while insufficient coverage will limit the extent of interfacial interactions, thereby restricting the charge separation pathways. Thus, precise control over growth parameters is critical to optimizing heterojunction functionality.

While methods like spin-coating, hydrothermal synthesis, and electrochemical deposition have been used to fabricate WO_3_/BiVO_4_ heterojunctions, they often yield nonuniform films, poor interfacial contact, or face scalability limitations.^3^ Aerosol-assisted chemical vapor deposition (AACVD) emerges as a promising alternative, offering scalability and the ability to produce high-quality thin films with controlled morphology and composition.^17,18^ AACVD can controllably deposit thin films over large areas by atomizing the precursor solution into fine aerosols, and can precisely adjust film properties through parameters such as precursor concentration and gas flow rate.^19^ However, systematic studies linking key AACVD parameters (e.g., precursor volume and deposition position within the reactor) to the performance of WO_3_/BiVO_4_ heterojunctions are scarce.^20,21^ Addressing this gap, this study employs AACVD to fabricate structurally controlled WO_3_/BiVO_4_ heterojunctions. Crucially, we adopt an inverted architecture (WO_3_ on top of BiVO_4_), diverging from the conventional configuration where WO_3_ is interfaced to the electrode.^3,11,12^ This design fully exploits the unique permeable nanofibrous morphology of WO_3_ deposited via AACVD, enabling simultaneous light transmission to the underlying BiVO_4_ and electrolyte penetration to the heterojunction interface. This overcomes limitations associated with conventional dense overlayer structures.

Our primary objectives were 2-fold: (1) to investigate the effects of WO_3_ precursor volume (10–40 mL) and spatial position within the deposition chamber relative to the precursor inlet point (inlet, mid, outlet) on the morphology, crystallinity, and optical properties of heterojunction thin films. Based on these findings, we aim to deposit heterojunction films featuring gradually varying structural characteristics. (2) To establish the correlation between these structural characteristics and the photoelectrochemical (PEC) water splitting performance, specifically the photocurrent density and incident photon-to-current efficiency (IPCE). This work advances the understanding of WO_3_/BiVO_4_ heterojunctions and provides valuable design principles for tailoring the AACVD process for energy conversion devices. By bridging the gap between synthesis control and functional performance, it offers novel insights for designing next-generation photoanodes with enhanced solar-to-hydrogen (STH) conversion efficiency,^22,23^ contributing to more sustainable hydrogen production as the world approaches the net-zero era.

## 2 Experimental Section

### 2.1 Film Fabrication

#### 2.1.1 BiVO_4_ Thin Film

BiVO_4_ thin films were deposited on FTO-coated glass substrates (NSG TEC 15, dimensions: 15 cm × 4.5 cm) using AACVD (Figure S1). Following a literature procedure, a precursor solution was prepared by dissolving 0.044 g of triphenyl bismuth (98%) and 0.028 g of vanadyl acetylacetonate (98%) in a mixed solvent of 15 mL acetone and 5 mL methanol.^24^ The mixture was ultrasonicated for at least 3 min until complete dissolution of the solid precursors was achieved. For samples intended as working electrodes in photoelectrochemical (PEC) testing, a thin quartz coverslip was used to mask a 0.4 × 1.2 cm strip along one edge of the FTO substrate during deposition, ensuring good electrical contact with the electrode clip during subsequent measurements. The substrate was placed in a reactor and preheated to 400 °C for a minimum of 15 min. The precursor solution was then atomized using an ultrasonic nebulizer, with compressed air serving as the carrier gas with mass flow controller (MFC) at a flow rate of 1000 sccm. The resulting aerosol was transported into the reactor for approximately 20 min until the entire precursor solution was consumed. BiVO_4_ films typically grow uniformly over the majority of the substrate area, eliminating concerns about the influence of sample position on film properties. Subsequently, the reactor temperature increased to 500 °C, and the sample was annealed in ambient air for over 2 h until the surface color turned from dark yellow to light yellow. The film was then allowed to cool under static room air to room temperature.

#### 2.1.2 WO_3_Thin Film

WO_3_ thin films were similarly deposited on FTO-coated glass substrates (NSG TEC 15, dimensions: 15 cm × 4.5 cm) via AACVD. Following a literature procedure, a precursor solution was prepared by dissolving 0.090 g of tungsten hexacarbonyl (97%) in a mixed solvent consisting of 20 mL acetone and 10 mL methanol [18]. The mixture was subjected to ultrasonication for at least 3 min to ensure complete dissolution of the solid precursor.

The substrate was loaded into the reactor and preheated to 375 °C for a minimum of 15 min. The precursor solution was then atomized using an ultrasonic nebulizer, with nitrogen serving as the carrier gas at a controlled flow rate of 1 L/min. The generated precursor aerosol was continuously delivered into the reactor until complete consumption of the precursor solution. Based on their position within the reactor, the samples were divided into inlet, mid, and outlet sections. The corresponding WO_3_ films were labeled as WO_3_-30 inlet, WO_3_-30 mid, and WO_3_-30 outlet, respectively (the WO_3_ mentioned below refers to WO_3_-30 mid by default). Subsequently, the reactor temperature was elevated to 500 °C, and the sample was annealed in ambient air for over 2 h until the film surface turned from dark blue to light gray. The film was then allowed to cool naturally to room temperature.

#### 2.1.3 Heterojunction Films

The WO_3_/BiVO_4_/FTO heterojunctions were fabricated through sequential depositions, where BiVO_4_ was first deposited on FTO substrates followed by WO_3_ deposition on top. To examine the influence of precursor quantity, the volume of the WO_3_ precursor solution transferred was systematically varied (10 mL, 20 mL, 30 mL, or 40 mL) while maintaining the same concentration of tungsten hexacarbonyl precursor (i.e., 8.53 mM) and ratio of solvents (i.e., 2:1 acetone: methanol). The resulting heterojunction films were correspondingly labeled as WO_3_-10/BiVO_4_, WO_3_-20/BiVO_4_, WO_3_-30/BiVO_4_, and WO_3_-40/BiVO_4_. For spatial characterization across the substrate, samples were additionally categorized based on their position in the deposition chamber relative to the gas inlet. Using WO_3_-30/BiVO_4_ as a representative example, samples near the precursor gas inlet were designated WO_3_-30/BiVO_4_-inlet, those in the central region as WO_3_-30/BiVO_4_-mid, and those near the gas outlet as WO_3_-30/BiVO_4_-outlet (Figure S1). The experimental approach ensured precise control over deposition parameters while allowing for detailed analysis of variations in film properties due to the spatial position in the deposition chamber.

### 2.2 Physical Characterization

The structural characterization of the materials was performed using grazing-incidence X-ray diffraction (GIXRD) patterns collected on a Panalytical Empyrean Thin Film PXRD system with a scanning range of 10–70° (step size: 0.05°, counting time: 0.5 s per step). A Cu K_α_ radiation source (λ = 1.5406 Å) operating at 40 kV and 40 mA was employed, with the incident beam angle fixed at 1.0°. For morphological analysis, the thin films were examined using scanning electron microscopy (SEM) with both JEOL JSM-7600 and JEOL JSM-IT700HR instruments. Prior to SEM preparation, the samples were cut into 1 cm × 1 cm pieces and coated with a layer of gold for approximately 10 s using an AGB7341 sputter coater. Optical properties were evaluated through UV–vis-NIR spectroscopy in transmission mode (200–800 nm wavelength range) using a Shimadzu UV-3600i Plus spectrophotometer.

The transient absorption spectroscopy setup incorporated a Nd:YAG laser system (Big Sky Laser Technologies, Ultra CFR Nd:YAG laser, 355 nm third harmonic, 6 ns pulse width) operating at 0.7 Hz. The laser fluence was adjusted to 150 μJ cm^– 2^ using neutral density filters (Comar Instruments). A 100 W Bentham IL1 tungsten lamp with grating (OBB-2001, Photon Technology International) served as the probe light source. The transmitted probe light was filtered through multiple long-pass and band-pass filters (Comar Instruments) to eliminate scattered laser light. Detection was achieved using a silicon photodiode (Hamamatsu S3071), with the signal processed through an amplifier (Costronics) and recorded on two time scales: μs-ms range using an oscilloscope (Tektronix TDS 2012c) and ms-s range via a DAQ card (National Instruments, NI USB-6211). Each transient absorption decay curve was obtained by averaging over 100 laser pulses. All data acquisition was performed using custom-developed software on the LabVIEW platform.

### 2.3 PEC Testing and Calculations

The PEC performance measurements were conducted using a three-electrode system housed in a quartz-walled electrochemical cell, with all samples uniformly cut to 1.5 cm × 1.2 cm dimensions. The three-electrode configuration consisted of the synthesized heterojunction thin film as the working electrode, a commercial Ag/AgCl/saturated-KCl reference electrode (Metrohm), and a platinum mesh counter electrode. The electrolyte was a pH 7 buffer solution prepared by mixing 2 M K_2_HPO_4_ and 2 M KH_2_PO_4_. Illumination was provided by an ozone-free xenon lamp (64 W, Hamamatsu) equipped with a KG3 filter, with the light intensity precisely measured using an optical power meter (PM 100, Thorlabs) and power sensor (S120UV, Thorlabs). Potential application and current measurement were performed using an Autolab potentiostat (PGSTAT12 with FRA2 module), maintaining optimal experimental conditions including minimal UV absorption through the quartz cell, stable pH environment via concentrated phosphate buffer, and controlled illumination with accurate intensity quantification. The system configuration enabled comprehensive electrochemical characterization while ensuring reproducible evaluation of photoelectrode performance under standardized testing conditions.

Applied potentials were converted to the reversible hydrogen electrode (RHE) using the Nernst equation:

1 $$V_{RHE}=V_{Ag/AgCl}+E_{Ag/AgCl}+0.059\times pH$$

Where *V_A_*_g/*A*g*Cl*_ is the experimentally measured voltage (vs Ag/AgCl saturated-KCl reference electrode), *E_A_*_g/_ *_A_*_g_ *_Cl_* is the standard potential of the Ag/AgCl saturated-KCl reference electrode, which is +0.197 V, and 0.059 is the coefficient of the Nernst equation at 25 °C. Photocurrent density was determined from the measured photocurrent and sample area:

2 $$J_{ph}=\frac{I}{A}$$

where *I* is the measured current and is the illuminated area of the sample. And the incident photon-to-current efficiency (IPCE) was calculated using the following equation:

3 $$IPCE\left(\lambda \right)=\frac{J_{ph}\left(A/cm^{2}\right)\;\times \;1240}{P_{in}\left(W/cm^{2}\right)\;\times \;\lambda \left(nm\right)}\times 100\%$$

where *P*_*in*_ is the incident monochromatic light power density, λ is the wavelength of the monochromatic light, and 1240 is a constant derived from the photon energy equation.

## 3 Results and Discussion

### 3.1 Film Appearance

The individual BiVO_4_ layer on FTO formed clear and light yellow films, while the individual WO_3_ layer on FTO formed semiopaque and white films. Each layer in the heterojunction films was deposited sequentially, with the subsequent layer being deposited only after the complete deposition and annealing process of the previous layer. The heterojunction samples formed semitransparent pale-yellow films. The film transparency gradually decreased with increasing precursor dosage. Notably, the film’s appearance also varies across different positions on a single substrate. The region near the center of the sample exhibited a uniform light yellow color, while the edges gradually faded. Due to the design of the reactor, the carrier gas flow does not uniformly cover the entire substrate surface as it travels toward the outlet. Consequently, regions near the substrate edges along its width remain uncoated, as illustrated in Figure S1. To ensure consistency and reliability of the results, only samples from the main growth region were selected for subsequent characterization and testing.

### 3.2 Crystal Structure and Chemical Composition

GIXRD was employed to characterize the crystal structures of the single-layer WO_3_, BiVO_4_, and heterojunction films deposited on FTO (Figure 1a). The diffraction patterns of the single-layer films confirm the presence of only monoclinic scheelite-structured BiVO_4_ and monoclinic WO_3_ on the FTO substrate. The primary characteristic peaks of monoclinic scheelite BiVO_4_ appear at 18.8° and 28.9°, corresponding to crystal growth along the (110) and (121) planes, respectively. Previous reports indicate that monoclinic scheelite BiVO_4_ exhibits superior photocatalytic activity compared to other polymorphs.^25^

**Figure 1:**
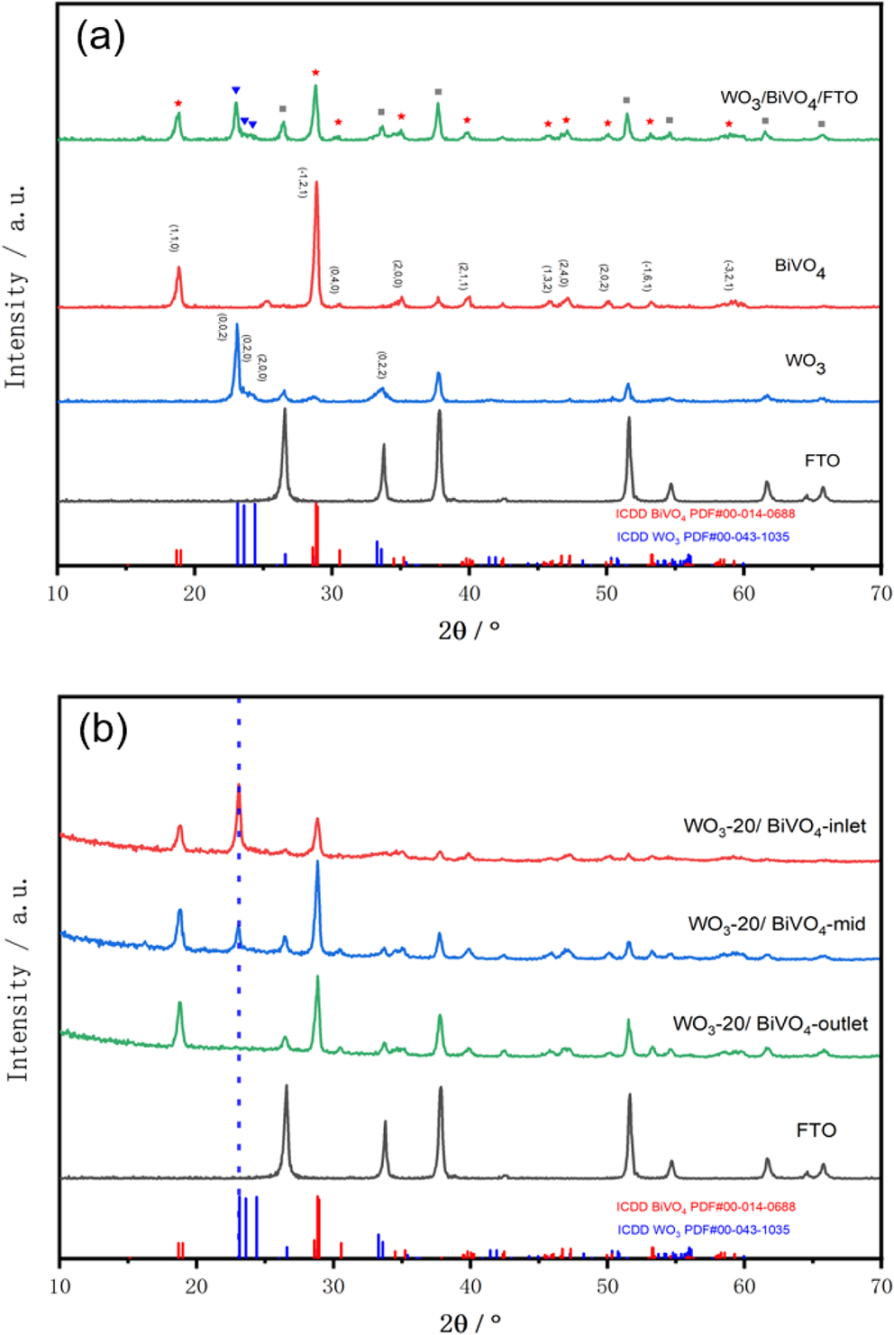
Grazing-incidence X-ray diffraction (GIXRD) patterns of (a) BiVO_4_, WO_3_, and WO_3_-30/BiVO_4_-mid films, and (b) exemplar position-dependent WO_3_-20/BiVO_4_ heterojunction samples.

For WO_3_, the monoclinic form shows the main characteristic peaks at 23.2° (002), 23.6° (020), and 24.4° (200). Our experimental pattern shows a strong peak at 23.1°, in line with the (002) plane, and suppressed growth in the following two planes. Both texture coefficient calculations (TC _(002)_ = 1.71) and Harris method (POF _(002)_ = 2.50) suggest preferential crystal growth along the (002) plane within the 23.1°–24.4° range, which enhances the diffraction intensity of the (002) plane while suppressing the (020) and (200) planes. This phenomenon has been documented in other studies on WO_3_.^4,26^

In the heterojunction sample’s diffraction pattern, all characteristic peaks align with those of the single-layer BiVO_4_ and WO_3_ parent films. Comparative analysis reveals that the intensity of the WO_3_ (002) peak at ∼23.1° varies depending on the sample’s position within the reactor. Taking WO_3_-20/BiVO_4_ as an example (Figure 1b), the sample located at the reactor’s inlet exhibits a distinct characteristic peak of monoclinic WO_3_ at 23.1°. In contrast, the peak intensity weakens for samples from the middle section of the reactor and becomes undetectable for those at the outlet, which is consistent with the trend reported in previous research.^20^ The gradual decrease in the WO_3_ (002) peak intensity from the inlet to the outlet region is attributed to the transformation of the crystal morphology from rod-like to nanofiber-like structures, as well as to variations in nanofiber density (Figure 3). As the substrate position approaches the outlet, the number of nanofiber-permeable structures grown on BiVO_4_ correspondingly decreases.

The surface composition of the WO_3_-30/BiVO_4_ mid heterojunction was investigated by high-resolution X-ray photoelectron spectroscopy (XPS), as shown in Figure 2. In the Bi 4f region (Figure 2a), two distinct peaks were observed at binding energies of 158.7 and 164.0 eV, corresponding to Bi 4f_7/2_ and Bi 4f_5/2_, respectively. These are characteristic of Bi^3+^ species in BiVO_4_, in agreement with previous reports.^27,28^ No additional peaks related to metallic Bi were detected, indicating good chemical stability of BiVO_4_ under X-ray exposure during analysis. In the W 4f region (Figure 2b), the W 4f_7/2_ and W 4f_5/2_ peaks were located at 35.5 and 37.6 eV, respectively, which are consistent with W^6+^ oxidation state in WO_3_.^11,29^ The absence of lower oxidation state features suggests complete oxidation of tungsten in the heterojunction. The well-defined and symmetric peak shapes with low background signal further support the formation of a chemically uniform WO_3_/BiVO_4_ interface.

**Figure 2:**
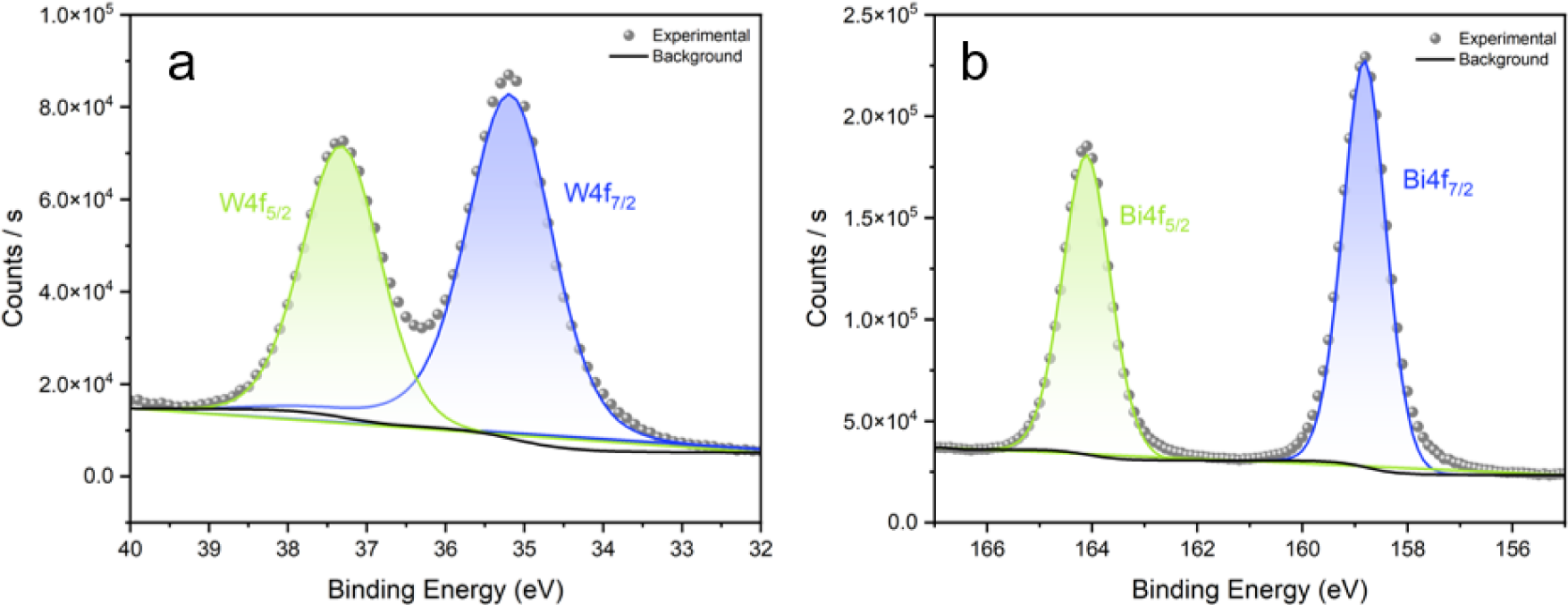
High resolution XPS spectra of (a) W 4f, (b) Bi 4f from the WO_3_–30/BiVO_4_-mid heterojunction films grown via AACVD (where the WO_3_ precursor volume of 30 mL was transferred and the sample was positioned in the middle of the deposition chamber).

### 3.3 Morphology

Figure 3 presents SEM images of single-layer BiVO_4_ and WO_3_ deposited on FTO. The BiVO_4_ film exhibits densely packed irregular-shaped crystals with grain sizes ranging from 100 to 200 nm, consistent with previously reported structures.^20^ For the WO_3_-inlet sample, the crystals display a rod-like morphology with root diameters of 100 nm and lengths of 300–400 nm, showing slight tapering at the tips. In contrast, both the mid and outlet sections demonstrate similar “grass-like” nanostructures composed of elongated nanofibers with diameters of 100–230 nm, though the outlet section exhibits comparatively shorter nanofibers.

Figure 4 illustrates the heterojunctions where WO_3_ crystals were grown on BiVO_4_. Figure 4 (a, b), (c, d), and (e, f) respectively show the inlet, mid, and outlet sections of samples prepared with 20 mL of WO_3_ precursor (i.e., samples WO_3_-20/BiVO_4_-inlet, WO_3_-20/BiVO_4_-mid and WO_3_-20/BiVO_4_-outlet, respectively). The BiVO_4_ film maintains a consistent thickness of 150–200 nm, with WO_3_ crystals preferentially growing along the edges of BiVO_4_ grains, resulting in either rod-like or fibrous structures. The inlet layer clearly displays a multistacked rod-like WO_3_ structure (similar to those seen in standalone WO_3_ films in Figure 3b) above both the FTO and BiVO_4_ layers. Comparative analysis between mid and outlet sections reveals fully developed nanofibers in the mid region versus nascent nanofiber growth in the outlet region. The morphological characteristics of the WO_3_ layer and the BiVO_4_ layer have been reported separately and are consistent with the morphological characteristics of the present samples,^14,30^ but there are few studies of heterojunction growth of WO_3_ nanofibers on the upper layer (i.e., an inverted heterostructure). The grass-like WO_3_ nanofibers (diameter: 100–230 nm) in mid/outlet regions create interwoven porous networks (Figure 4c–f). This architecture provides dual pathways: (i) vertical light channels enabling >50% transmittance at 515 nm (discussed later), and (ii) open pores for electrolyte diffusion to underlying BiVO_4_ OER sites.

**Figure 3:**
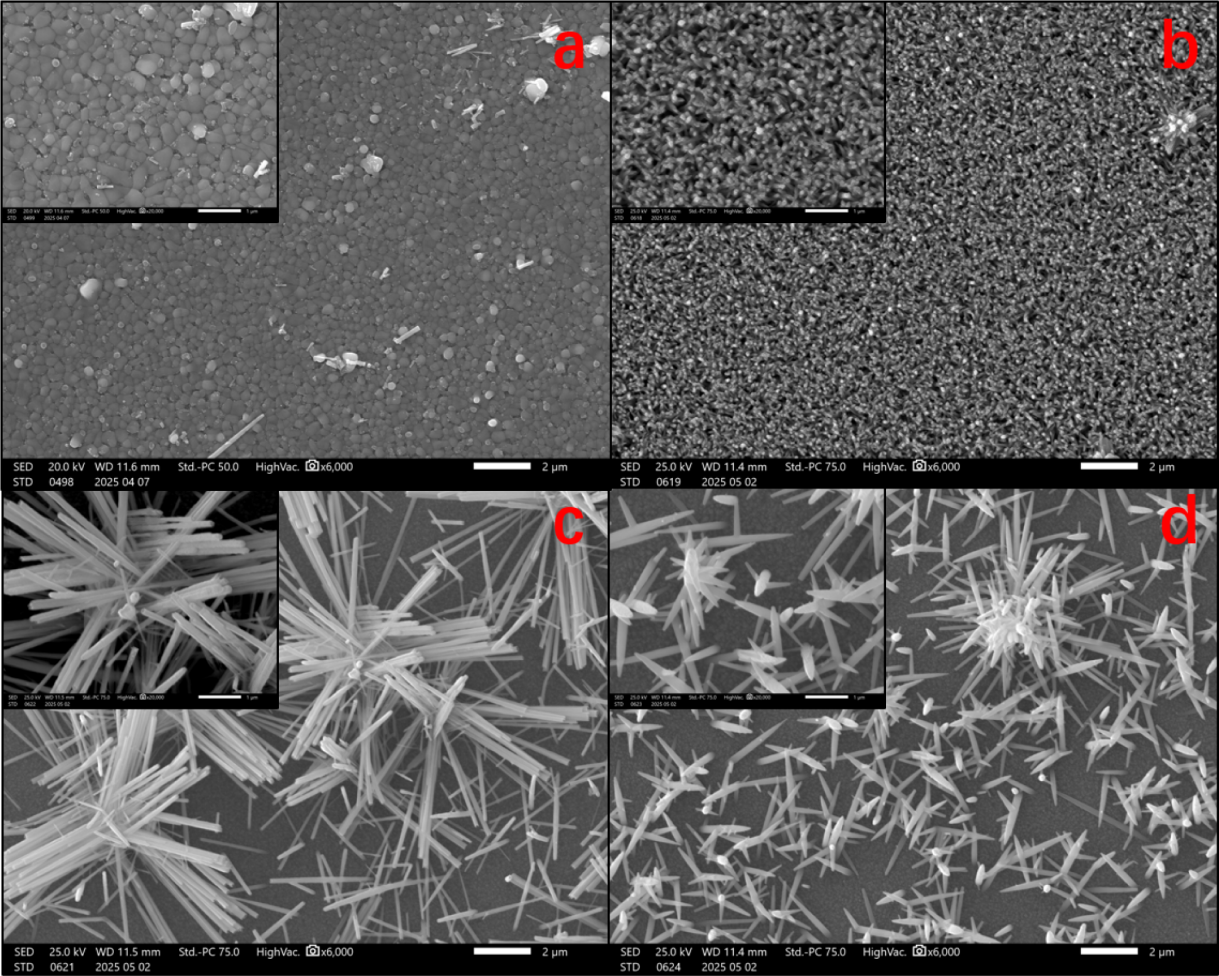
Scanning electron microscopy (SEM) images of single-layer (a) BiVO_4_, (b) WO_3_-inlet, (c) WO_3_-mid, and (d) WO_3_-outlet films.

**Figure 4:**
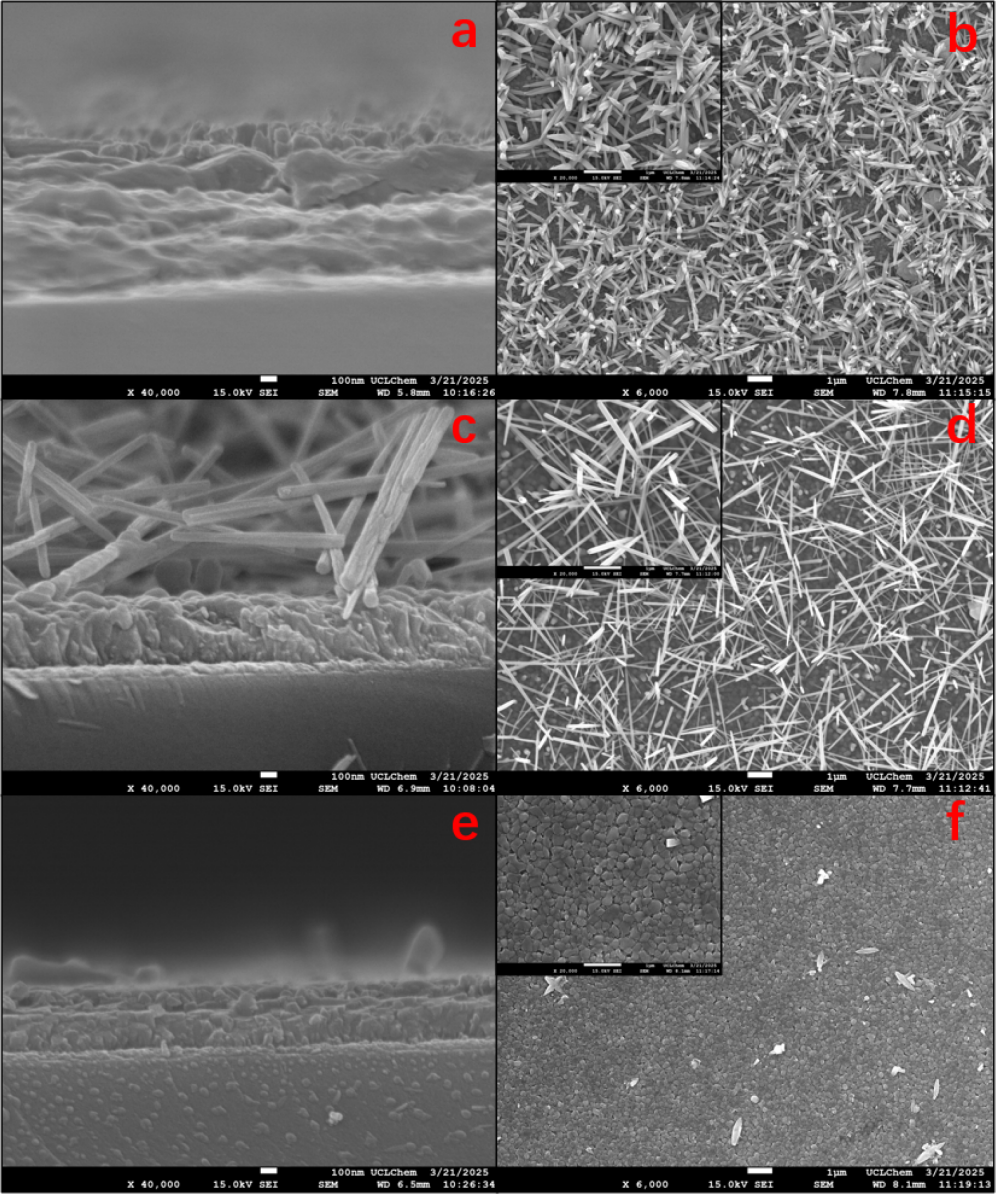
Cross-sectional and surface SEM images of WO_3_-20/BiVO_4_ heterojunction samples at (a, b) inlet, (c, d) mid, and (e, f) outlet regions.

Figure S2 compares midsection samples prepared with different precursor volumes: (a) WO_3_-20/BiVO_4_-mid, (b) WO_3_-10/BiVO_4_-mid, (c) WO_3_-30/BiVO_4_-mid, and (d) WO_3_-40/BiVO_4_-mid. The images demonstrate that increasing precursor volume extends deposition time and transforms the growth mode from isolated nanofiber clusters to complete BiVO_4_ surface coverage. Higher precursor quantities produce denser nanofiber networks with increased diameters (growing from initial 90 to 200 nm) and progressively sharper tips.

### 3.4 Optical Properties

The optical properties of the WO_3_-30/BiVO_4_-mid heterojunction and its parent materials (standalone BiVO_4_ and standalone WO_3_-mid) were investigated using UV–vis spectroscopy (Figure 5a). The single-layer WO_3_ film exhibited a steep absorption edge near 385 nm (corresponding to ∼3.66 eV), which is characteristic of the wide bandgap of WO_3_ and its limited ability to harvest visible light. In contrast, the main absorption edge of the BiVO_4_ film is red-shifted to ∼515 nm (corresponding to ∼2.56 eV), and thus shows significantly improved visible light harvesting.

**Figure 5:**
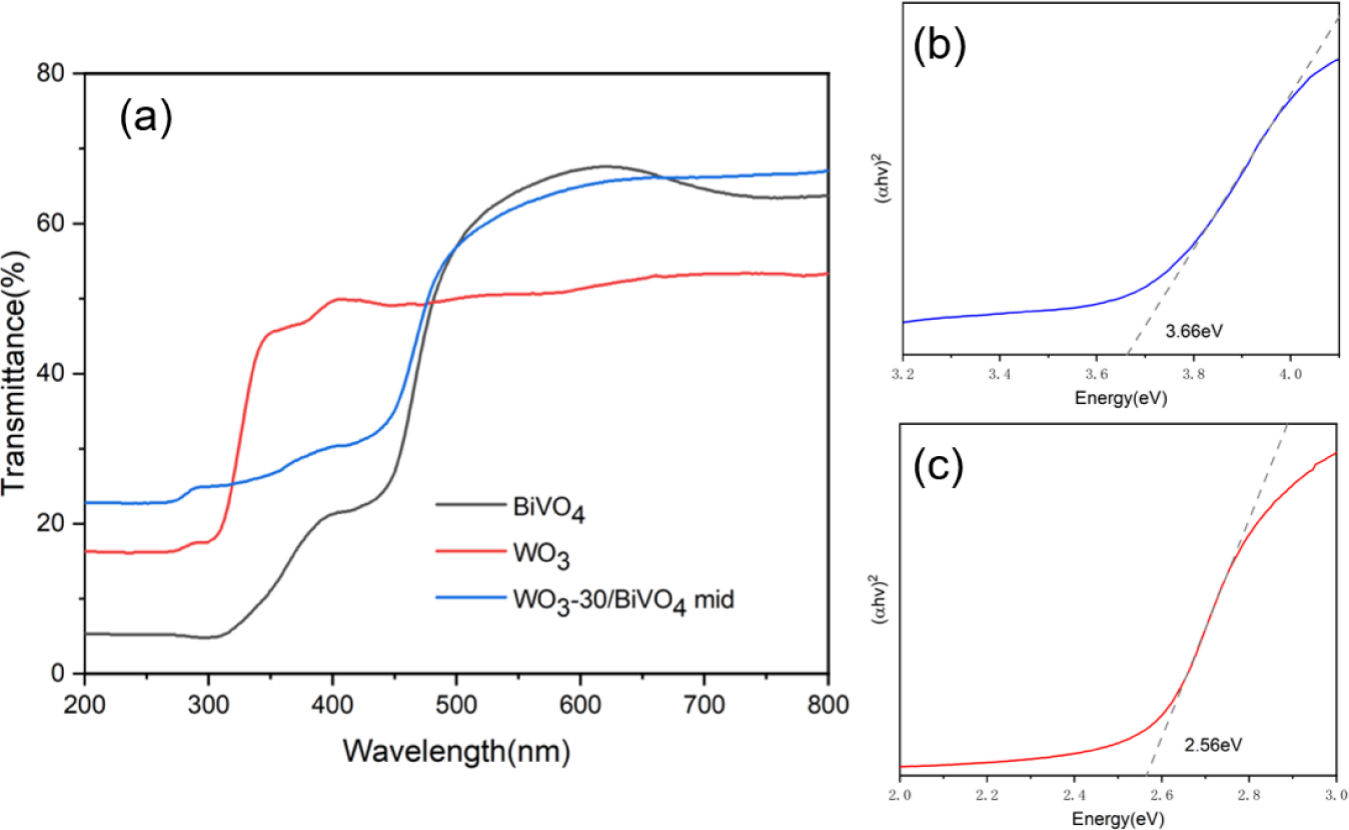
(a) UV–vis transmittance spectra of BiVO_4_, WO_3_, and WO_3_-30/BiVO_4_-mid heterojunction and (b, c) Tauc plots for bandgap determination of WO_3_ and BiVO_4_, respectively.

The transmittance curve of the heterojunction sample (WO_3_-30/BiVO_4_-mid) lies between those of the two individual materials, with its absorption edge positioned in an intermediate region between WO_3_ and BiVO_4_, indicating synergistic light absorption properties across the UV to visible range. Notably, no distinct additional absorption edge appears in the heterojunction spectrum, suggesting complete interfacial coverage and effective coupling between the WO_3_ and BiVO_4_ layers.

The optical bandgaps of WO_3_ and BiVO_4_ were calculated using the Tauc method (Figure 5b,c). WO_3_ exhibits an direct bandgap of ∼3.66 eV, consistent with its semiconductor properties, while BiVO_4_ shows a direct bandgap of ∼2.56 eV, matching reported data for monoclinic scheelite structures.^2,31^

### 3.5 PEC Performance and Carrier Dynamics

The anodic photocurrent responses of WO_3_ and BiVO_4_ in their oxidation of water is shown in Figure 6a. The WO_3_-30/BiVO_4_-mid heterojunction achieved a photocurrent density of 0.8 mA·cm^–2^ at 1.23 V_RHE_, significantly higher than the additive performance of single-layer WO_3_ (0.03 mA·cm^–2^) and BiVO_4_ (0.37 mA·cm^–2^), indicating that the heterojunction synergistically promotes charge separation. Furthermore, comparative studies were conducted on the three regions of the heterojunction film synthesized with 30 mL WO_3_ precursor, as well as the effects of varying precursor volumes (Figure S3a). At 1.23 V_RHE_, samples prepared with 10 mL, 20 mL, and 40 mL WO_3_ precursor solutions exhibited similar photocurrent densities (0.71 mA·cm^–2^), while the sample with 30 mL demonstrated the highest photocurrent density (0.82 mA·cm^–2^) (Figure S3b). Consequently, the three regions of the 30 mL precursor sample were further investigated.

**Figure 6:**
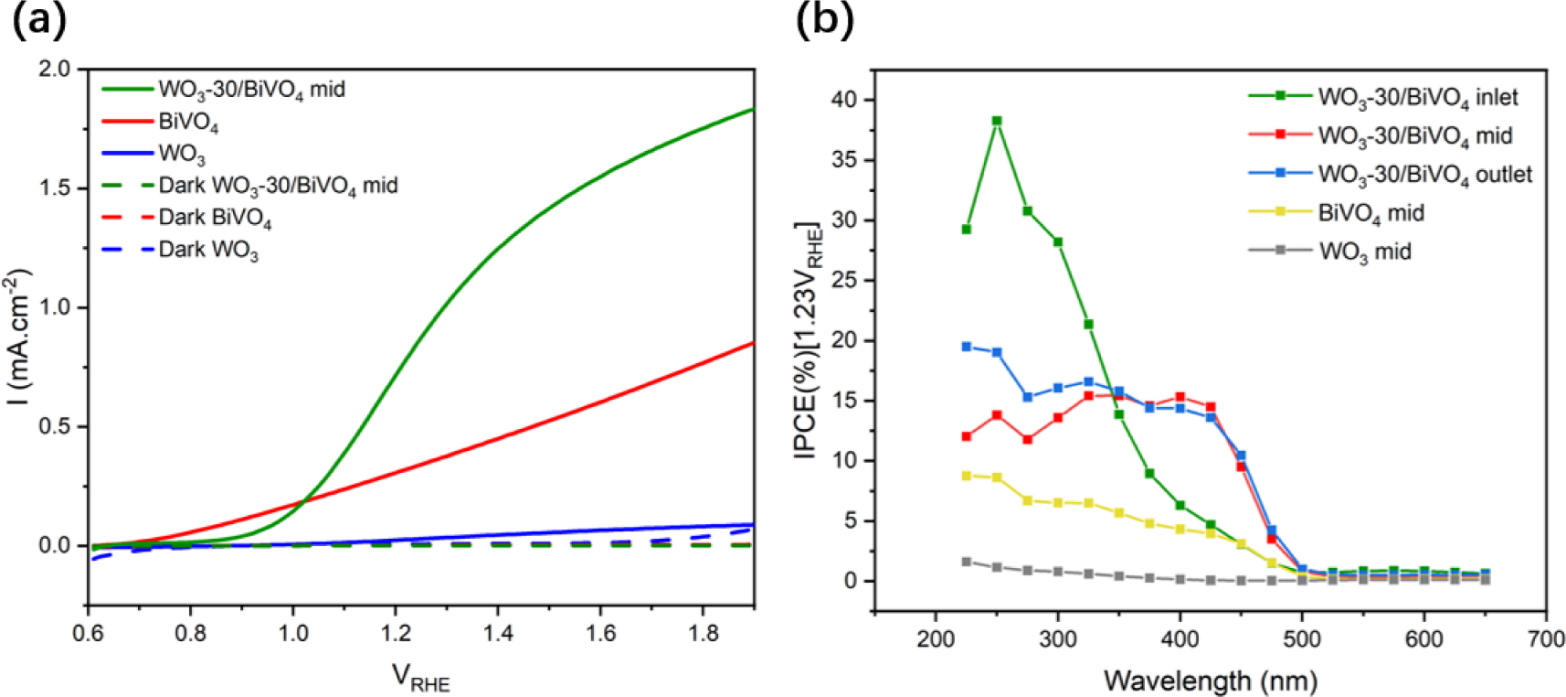
(a) Photocurrent density–voltage (J-V) curves of single-layer and heterojunction films, and (b) Incident photon-to-current efficiency (IPCE) spectra of WO_3_-30/BiVO_4_ heterojunction for the inlet, mid and outlet regions and single-layer BiVO_4_ and WO_3_.

The inlet region shows the weakest photocurrent density (0.32 mA·cm^–2^), which is consistent with the transmittance data of WO_3_-30/BiVO_4_-inlet (Figure S3a), confirming its poor photon absorption behavior. Additionally, the high-density rod-like structure in this region enhances light scattering, preventing the underlying BiVO_4_ from receiving sufficient photons and consequently hindering carrier excitation. In contrast, the mid and outlet regions exhibited higher photocurrent densities at 1.23 V_RHE_ (mid: 0.82 mA·cm^–2^, outlet: 0.65 mA·cm^–2^) compared to the inlet, demonstrating that the heterojunction improved charge separation efficiency, reduces recombination, and thus improves photocatalytic water oxidation. This can also explain why the sample using 30 mL WO_3_ precursor shows the highest photocurrent density, as this sample strikes a balance between transmittance through the WO_3_ layer and possessing a high contact area at the interface with BiVO_4_. However, the onset potential shifted to more anodic values. Between 0.6 V_RHE_ and 1.0 V_RHE_, the photocurrent density of the outlet sample increases the slowest, while that of the inlet sample rises the fastest. The slower photocurrent rise in nanofiber-rich regions (mid/outlet) stems from efficient electron transfer to WO_3_, requiring higher bias to drive water oxidation. Conversely, rod-dominated inlet regions exhibit faster onset due to limited heterojunction formation, but ultimately lower efficiency due to poor light harvesting.

To verify the light absorption and response characteristics of the samples, the incident photon-to-current conversion efficiency (IPCE) of single-layer BiVO_4_, single-layer WO_3_, and the three regions of the WO_3_-30/BiVO_4_ heterojunction sample were measured under a constant potential of 1.23 V_RHE_ across a wavelength range of 225–600 nm (Figure 6b). In the ultraviolet region, the inlet region sample exhibited IPCEs between 22%–38%, which sharply decreased to 5% beyond 350 nm. This observation aligns with our expectations, as the lower transmittance of WO_3_ in the inlet region hindered BiVO_4_ from absorbing sufficient photons, leaving only the wide-bandgap WO_3_ layer to contribute significantly to the observed activity. The mid and outlet regions demonstrated similar IPCE performance. The nanofiber-structured WO_3_ minimally interfered with light absorption by the underlying BiVO_4_ layer, allowing the heterojunction structure to effectively enhance both the overall light-harvesting efficiency and the activity of surface catalytic sites. Consequently, these regions showed higher IPCE values than single-layer BiVO_4_ across both UV and visible light regions.

To evaluate the stability of the WO_3_/BiVO_4_ heterojunction, the chronoamperometry was measured under illumination at 1.23 V_RHE_ for a duration of 3600 s in the same electrolyte configuration (Figure S4). The photoresponse exhibits an initial rapid decay. The current density sharply drops from 1.65 mA·cm^–2^ at *t* = 0 s to 0.90 mA·cm^–2^ within the first 360 s. This corresponds to a 45% loss of the initial photocurrent. Subsequently, the decay rate significantly slows down. From 360 to 1800 s, the current density only decreases by 0.19 mA·cm^–2^ (from 0.90 mA·cm^–2^ to 0.71 mA·cm^–2^). In the final stage (from 1800 to 3600 s), the decay rate further attenuates, with the current density dropping by only 0.13 mA·cm^–2^ to reach a final stable value of 0.58 mA·cm^–2^. Following multiple photoresponse tests, totaling approximately 20 h, the post-tested sample was characterized by X-ray Diffraction (XRD), as shown in Figure S5. Compared to the original XRD pattern (Before), the characteristic peaks of WO_3_ (marked with blue inverted triangles, ∇) exhibit a notable reduction in intensity in the posttest sample (After). This reduction in WO_3_ crystallinity or material quantity is likely attributed to the cumulative effect of repeated mechanical handling (including repeated immersion and removal from the buffer solution), potential photocorrosion under sustained bias, and structural degradation such as attrition, agglomeration, or collapse of the WO_3_ nanostructure.

Electrochemical impedance spectroscopy (EIS) measurements were conducted to evaluate the photoelectrochemical (PEC) properties of the single-layer and heterojunction electrodes. Figure 7a shows the Nyquist plots of BiVO_4_, WO_3_, and WO_3_/BiVO_4_ photoanodes measured under illumination at 1.23 V_RHE_. The inset illustrates the equivalent circuit (R_s_–CPE–R_ct_) used to fit the impedance data, where R_s_ represents the series resistance, R_ct_ denotes the interfacial charge-transfer resistance, and CPE accounts for nonideal capacitive behavior of the electrode surface. For the single-component electrodes, the Nyquist plot of WO_3_/FTO (blue curve) exhibits an almost vertical line along the Z′ axis, suggesting a predominantly capacitive response with a very fast charge-transfer process, where no distinct semicircle is observed. This feature is consistent with the superior bulk charge transport property and high donor density (N_D_) of WO_3_ confirmed by Mott–Schottky analysis (Figure 7b). In contrast, the BiVO_4_/FTO electrode (red curve) displays a pronounced semicircle with a fitted R_ct_ of approximately 427.5 Ω and a characteristic frequency of 5.18 Hz. The large R_ct_ value reveals the intrinsic limitation of BiVO_4_, mainly due to sluggish hole-transfer kinetics and severe bulk/surface charge recombination. Upon coupling WO_3_ with BiVO_4_ to form a heterojunction (green curve), the semicircle diameter decreases significantly, and the fitted R_ct_ drops to 276.2 Ω—a 35.3% reduction compared to bare BiVO_4_. Meanwhile, the peak frequency markedly increases from 5.18 to 115.25 Hz, indicating a substantial enhancement in interfacial charge-transfer kinetics. The reduced R_ct_ and higher characteristic frequency confirm that the acceleration of carrier transport while suppressing bulk charge recombination. This improvement is attributed to the formation of an internal electric field at the WO_3_/BiVO_4_ interface, as evidenced by the positive shift in the flat-band potential (E_fb_) from the Mott–Schottky analysis (Figure 7b). Overall, the EIS results quantitatively demonstrate that the WO_3_ layer acts as both an efficient electron transport channel and a hole-transfer promoter, which is key to the superior photoelectrochemical performance of the WO_3_/BiVO_4_ heterojunction.

**Figure 7:**
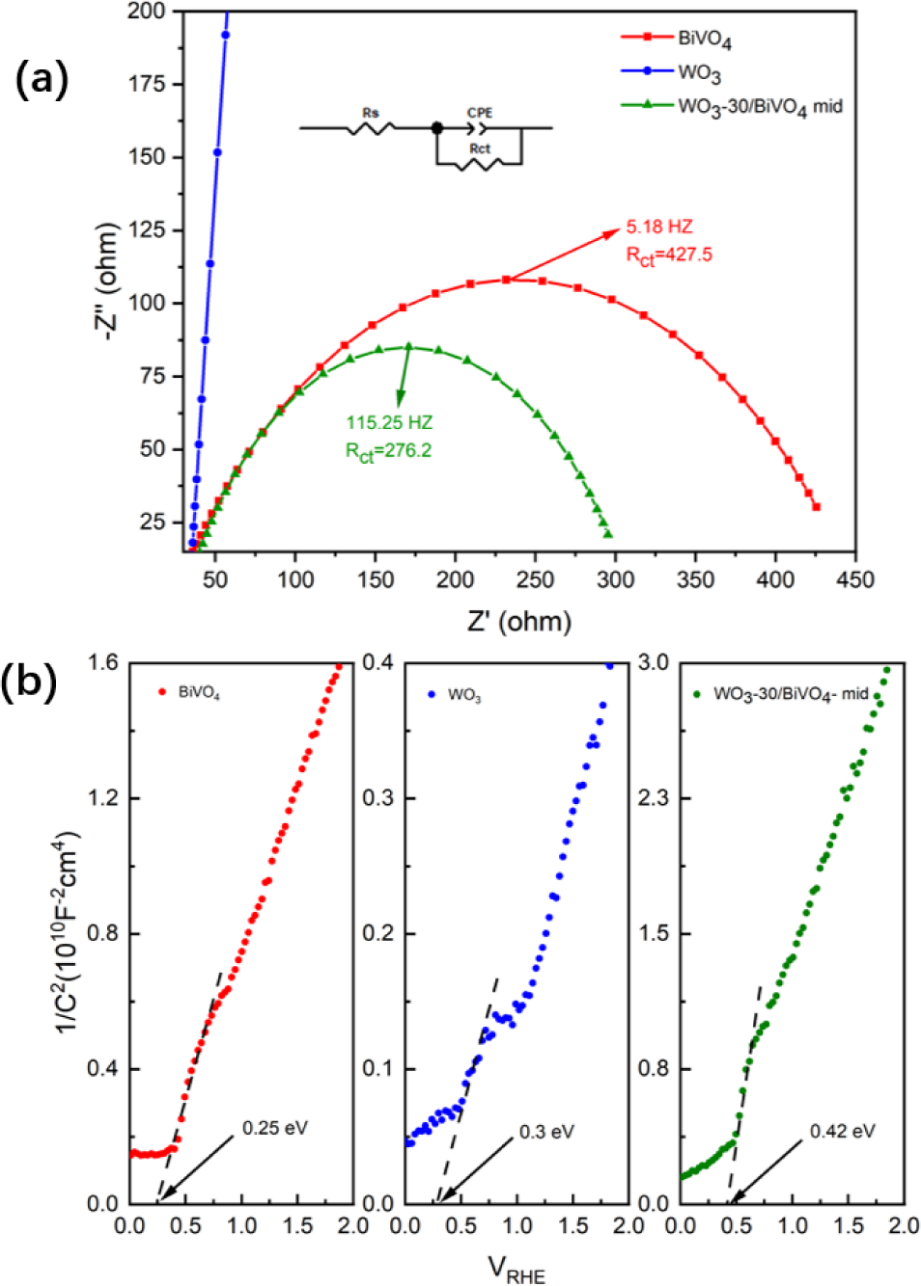
(a) Nyquist plots of BiVO_4_, WO_3_, and WO_3_/BiVO_4_ photoanodes measured under illumination at 1.23 VRHE. (b) Mott–Schottky plots of BiVO_4_, WO_3_, and WO_3_/BiVO_4_ photoanodes measured in the dark.

The corresponding Mott–Schottky plots (1/C^2^ vs V_RHE_) are shown in Figure 7b. All three electrodes display positive slopes in their linear regions, confirming their n-type semiconductor behavior, where the measured capacitance is primarily governed by the depletion layer. The E_fb_ values were extracted by extrapolating the linear portion of the plots to the potential axis (1/C^2^ = 0). The flat-band potentials of the single-component electrodes were determined to be 0.25 V_RHE_ for BiVO_4_/FTO and 0.30 V_RHE_ for WO_3_/FTO. A comparison of the slopes provides insight into the donor densities, since the slope is inversely proportional to N_D_ (slope ∝ 1/N_D_). The slope of the WO_3_/FTO electrode (≈ 0.35 × 10 ^1 0^ F^–2^ ·cm^4^ ·V^–1^ within 0.5–0.8 V) is notably smaller than that of BiVO_4_/FTO (≈ 1.1 × 10^–1 0^ F ^2^ ·cm^4^ ·V^–1^ within 0.4–0.9 V). This difference indicates that WO_3_ possesses a higher intrinsic donor density than BiVO_4_, supporting its advantageous role in electron collection and transport within the composite system.

For the WO_3_/BiVO_4_/FTO heterojunction electrode (with WO_3_ as the outer layer), the E_fb_ shifts positively to 0.42 V_RHE_, corresponding to a 0.12 V anodic shift compared with single-layer WO_3_. This positive displacement is commonly attributed to interfacial dipole formation or favorable band bending upon junction formation between WO_3_ and BiVO_4_. Considering the measured band gaps of 3.66 eV for WO_3_ (Figure 5b) and 2.56 eV for BiVO_4_ (Figure 5c), the valence band maximum (E_VB_) and conduction band minimum (E_CB_) positions can be estimated. Taking the E_fb_ values as an approximation of E_CB_ for n-type semiconductors, the corresponding E_VB_ values are approximately 3.96 V_RHE_ for WO_3_ and 2.81 V_RHE_ for BiVO_4_. This establishes a staggered type-II band alignment, in which the conduction band of BiVO_4_ lies above that of WO_3_, and its valence band lies below. Consequently, upon illumination, photogenerated electrons in BiVO_4_ can readily transfer to WO_3_, while holes remain in BiVO_4_. This charge redistribution strengthens the space-charge region and enhances the built-in electric field within the WO_3_ layer. Because WO_3_ is in direct contact with the electrolyte, a more positive external potential is required to flatten its energy bands. The observed E_fb_ of 0.42 V thus provides direct evidence of interfacial band bending, which facilitates efficient separation and transport of photogenerated charge carriers across the BiVO_4_/WO_3_ junction, ultimately contributing to the improved PEC performance of the heterostructure electrode.

The charge carrier dynamics was studied using transient absorption spectroscopy (TAS). Figure 8 compares the transient absorption decay signals of the samples at probed at 550 nm under an applied potential of 1.23 V_RHE_ in 2 M K_2_HPO_4_ and KH_2_PO_4_ buffer. Previous studies of WO_3_ and BIVO_4_ have shown that hole carriers absorb most prominently in the blue region of the electromagnetic spectrum (i.e., centered at ∼500 nm in WO_3_ ^32,33^ and ∼ 550 nm in BiVO_4_ ^34^). The TAS results showed that the single-layer WO_3_ exhibits minimal long-lived hole carrier signals, which are required to drive water oxidation on this material,^35^ and is in agreement with the low IPCE seen herein for ∼ 355 nm excitation of this material (∼1%; Figure 6b). In contrast, the heterojunction sample WO_3_-30/BiVO_4_-mid demonstrates nearly double the initial hole carrier signal compared to single-layer BiVO_4_. As both the single-layer BiVO_4_ and WO_3_-30/BiVO_4_-mid heterojunction samples showed a similar degree of light absorption at 355 nm (Figure 5a), this was clear evidence for enhanced charge carrier separation in this heterojunction system. Importantly, the heterojunction structure significantly prolongs hole carrier lifetime to the post ms time scale required for water oxidation on BiVO_4_.^36^ Again, these results were in line with the improved performance seen in our IPCE measurements at ∼355 nm excitation, where the heterojunction (∼15%) showed more than twice the IPCE of the single-layer BiVO_4_ material (∼7%).

**Figure 8:**
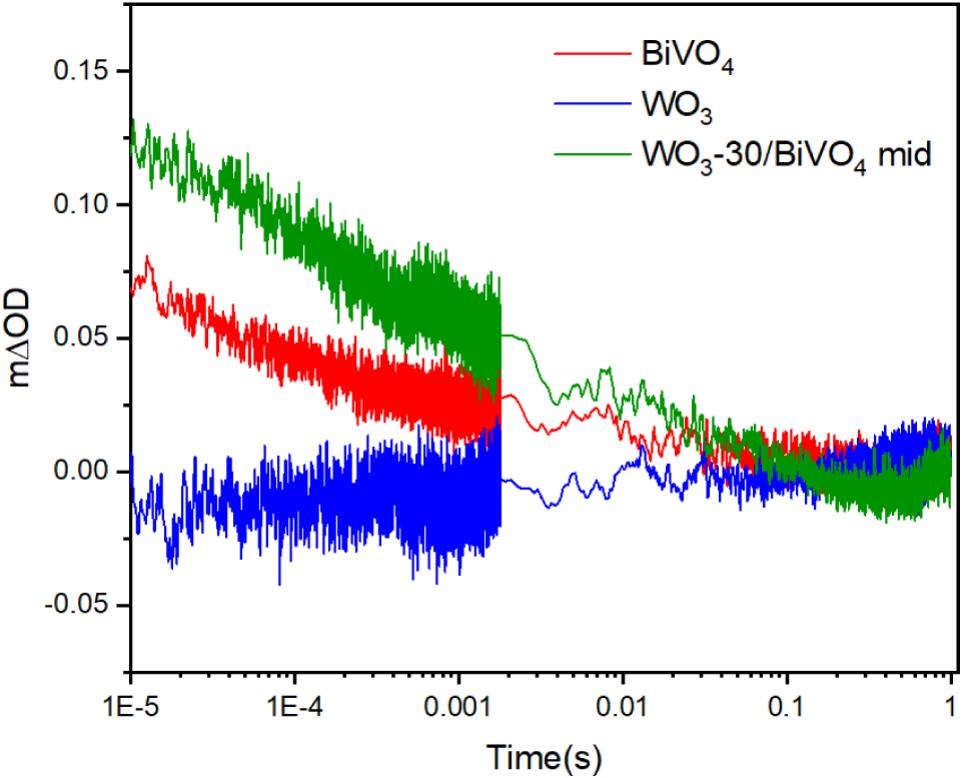
Transient absorption decay kinetics of BiVO_4_, WO_3_, and WO_3_-30/BiVO_4_-mid samples measured in 2 M K_2_HPO_4_ and KH_2_PO_4_ buffer (pH = 7) at 1.23 VRHE with a 550 nm probe wavelength using 355 nm laser pulse excitation.

#### 3.5.1 Comparisons with Literature

The solar predicted photocurrents observed herein (0.001–2.16 mA·cm^–2^ in 0.61–2.41 V_RHE_) are now compared with the literature (Table 1). It should be noted that all references cited in this chapter use the FTO-WO_3_–BiVO_4_ sequence, whereas this study employs the FTO-BiVO_4_–WO_3_ deposition sequence.

**Table 1:** Summary of the Synthesis, Microstructure, and Photocatalytic Water Oxidation Activity for Various WO_3_/BiVO_4_ Reported in the Literature

Synthesis	Microstructure of WO_3_	Photocurrent (mA·cm^–2^)	IPCE	sref
WO_3_: spin coating BiVO_4_: spin coating	Thin film	0.12 at 1 V vs Ag/AgCl	82% at 350 nm	^11^
WO_3_: flame vapor deposition BiVO_4_: drop casting	Nanowire and nanoparticle	0.7 at 1.23 V_RHE_	70% at 300 nm	^37^
WO_3_: sol–gel BiVO_4_: electrodeposition	Coral flake and nanoblocks	1.66 at 1.23 V_RHE_	-	^38^
WO_3_: hydrothermal process BiVO_4_: metal-organic precursor solution decomposition	Nanosheets and thin films	3 at 1.23 V_RHE_	40% at 450 nm	^39^
WO_3_: aerosol assisted chemical vapor deposition BiVO_4_: aerosol assisted chemical vapor deposition	Nnanoneedles and thin film	0.7 at 1.23 V_RHE_	-	^40^
This project	Nnanoneedles and thin film	0.8 at 1.23 V_RHE_	15% at 350 nm	

Chatchai et al. deposited WO_3_/BiVO_4_ composite films on FTO substrates by spin-coating. With the WO_3_ layer inserted between BiVO_4_ and FTO, a photocurrent of 0.12 mA·cm^–2^ at 1 V vs Ag/AgCl and an IPCE of 82% at 350 nm were achieved, attributed to effective charge carrier separation at the heterojunction interface.^11^ Liu et al. compared “sandwich-type” and “crossed-finger-type” multilayer WO_3_/BiVO_4_ heterojunctions. A photocurrent of 0.7 mA·cm^–2^ at 1.23 V_RHE_ demonstrated that the heterojunction structure directly impacts interface charge transfer efficiency.^37^ Fang et al. fabricated a ternary WO_3_/BiVO_4_/NiOOH heterojunction photoanode via sol–gel and electrodeposition, with coral-like BiVO_4_ flakes wrapping around WO_3_ nanorods and surface-modified NiOOH nanoparticles. They achieved a photocurrent density of 1.66 mA·cm^–2^ at 1.23 V_RHE_.^38^ Kim et al. synthesized BiVO_4_ nanosheets on WO_3_ nanorods (BiVO_4_–NLs/WO_3_–NRs) using a MOF-derived method. Under AM 1.5G illumination and at 1.23 V _RHE_, they attained a photocurrent density of 3 mA·cm^–2^, attributed to type II heterojunction charge separation and enhanced light scattering and reaction sites of the nanosheets.^39^ Creasey et al. prepared WO_3_/BiVO_4_ nanoneedles via AACVD, achieving a photocurrent density of 0.8 mA·cm^–2^ at 1.23 V_RHE_.^40^

Among the above studies, the work by Creasey et al. employed synthesis and testing conditions most similar to those used in this study, with the primary difference being the reversed deposition order of WO_3_ and BiVO_4_ in our case. Nevertheless, a comparable or slightly higher photocurrent density was achieved in this study. Compared to samples synthesized by other methods reported in the literature, the performance of the sample is lower than some. This can be attributed to the intrinsic issue of band alignment mismatch caused by the reversed WO_3_/BiVO_4_. However, despite this limitation, the sample still exhibits a pronounced photoresponse, indicating that this approach offers a potential pathway for constructing photocatalytic heterojunctions.

Based on the above analysis, future research should focus on two key strategies: interfacial band engineering and enhancement of surface catalytic activity. Specifically, introducing an ultrathin buffer layer (such as Fe_2_O_3_ or MoS_2_) between WO_3_ and BiVO_4_, or applying interfacial doping (such as W-doping of BiVO_4_ or Mo-doping of WO_3_), could precisely tune the band-edge alignment and construct a more favorable “stepwise” charge transfer pathway. Furthermore, to reduce the overpotential of the oxygen evolution reaction (OER) and suppress surface charge recombination, it is necessary to modify the top WO_3_ layer with suitable cocatalysts (such as NiOOH or FeOOH). By integrating these optimization strategies, the photoelectrochemical performance of the WO_3_/BiVO_4_ inverted heterojunction is expected to be significantly enhanced, potentially reaching or even surpassing the best values reported in the literature.

## 4 Conclusions

Aerosol-assisted chemical vapor deposition (AACVD) was successfully used to sequentially fabricate WO_3_/BiVO_4_ heterojunction films that exhibited significantly enhanced photoelectrochemical (PEC) water-splitting performance compared to their standalone WO_3_ or BiVO_4_ counterparts, demonstrating the synergy afforded by heterostructure engineering. This study strategically employed an inverted architecture (WO_3_ on top of BiVO_4_), capitalizing on the unique nanostructural capabilities afforded by the AACVD process. Precise control over deposition parameters proved paramount: increasing WO_3_ precursor volume systematically transformed the nanostructure from isolated nanofibers to denser networks, with volumes exceeding 40 mL leading to overgrown layers that diminished optical transparency without commensurate PEC gains. Furthermore, inherent aerosol concentration gradients within the reactor resulted in distinct position-dependent crystallinity and morphology of the WO_3_ layers with respect to the gas flow direction. Near the inlet, rod-like structures were seen, and at the mid and outlet positions finer “grass-like” nanofibers were seen.

The interplay between deposition parameters, nanostructure, and performance was studied. Samples from the midreactor region, fabricated with an optimal precursor volume (WO_3_-30/BiVO_4_-mid), achieved a standout photocurrent density of 0.82 mA·cm^–2^ at 1.23 V_RHE_, representing a 121% enhancement over single-layer BiVO_4_ (0.37 mA·cm^–2^) and WO_3_ alone (0.03 mA·cm^–2^). The nanofibrous WO_3_ overlayer, particularly in the midreactor region, enabled simultaneous efficient photon transmission to the underlying BiVO_4_ absorber and electrolyte penetration to the heterojunction interface, overcoming limitations of conventional dense overlayers. Transient absorption spectroscopy provided mechanistic insight, confirming prolonged charge carrier lifetimes within the heterojunction, which directly correlated with the observed performance enhancement. This tailored hierarchical structure therefore enhanced light scattering, extended the photon path length, and provided a higher surface area for interfacial charge transfer.

These findings underscore the necessity for meticulous control over AACVD parameters—specifically precursor volume and spatial positioning relative to reactor flow dynamics—to engineer WO_3_ nanostructures that maximize the functional benefits of the heterojunction. The work successfully redefines viable heterojunction configurations by demonstrating that a nanostructured, permeable WO_3_ overlayer, achievable through AACVD, can concurrently optimize light harvesting, charge separation, and mass transport. This provides a new avenue in the rational design of WO_3_/BiVO_4_ systems for solar-driven water splitting and provides practical guidelines for the scalable fabrication of high-performance photoanodes. Future efforts should focus on further enhancing operational stability and efficiency, potentially through targeted interface engineering or strategic cocatalyst integration.

## Supplementary Material



## Notes

Additional experimental details, AACVD setup schematics, precursor-dependent morphological comparisons, PEC performance data, complementary structural characterization, and representative chronoamperometry results (PDF)
