# Supplementary material for: Spatially Engineered WO3 Nanofibers on BiVO4: A Route to High-Efficiency Photoelectrochemical Water Splitting
Source: ACS Appl Energy Mater. 2025 Nov 25;8(23):17334–45. doi: 10.1021/acsaem.5c02686 (PMC12690508; doi:10.1021/acsaem.5c02686)
Supplement: Supplementary file 1 [file ae5c02686_si_001.pdf]

Supporting Information

Spatially Engineered WO_3 Nanofibers on $BiVO_4$: A Route to High-Efficiency Photoelectrochemical Water Splitting

Haochen Li,[a] *Zhipeng Lin,*[b] *Fei Guo,*[a] *Shuhui Li,*[a] *Andreas Kafizas,*[b] *Christopher S. Blackman,*[a] *Claire J. Carmalt,*[a*]

[a]Department of Chemistry, University College London, 20 Gordon Street, London, WC1H 0AJ, UK

[b]Department of Chemistry, Molecular Science Research hub, Imperial College London, London W12 0BA, UK

Corresponding author's email address：c.j.carmalt@ucl.ac.uk

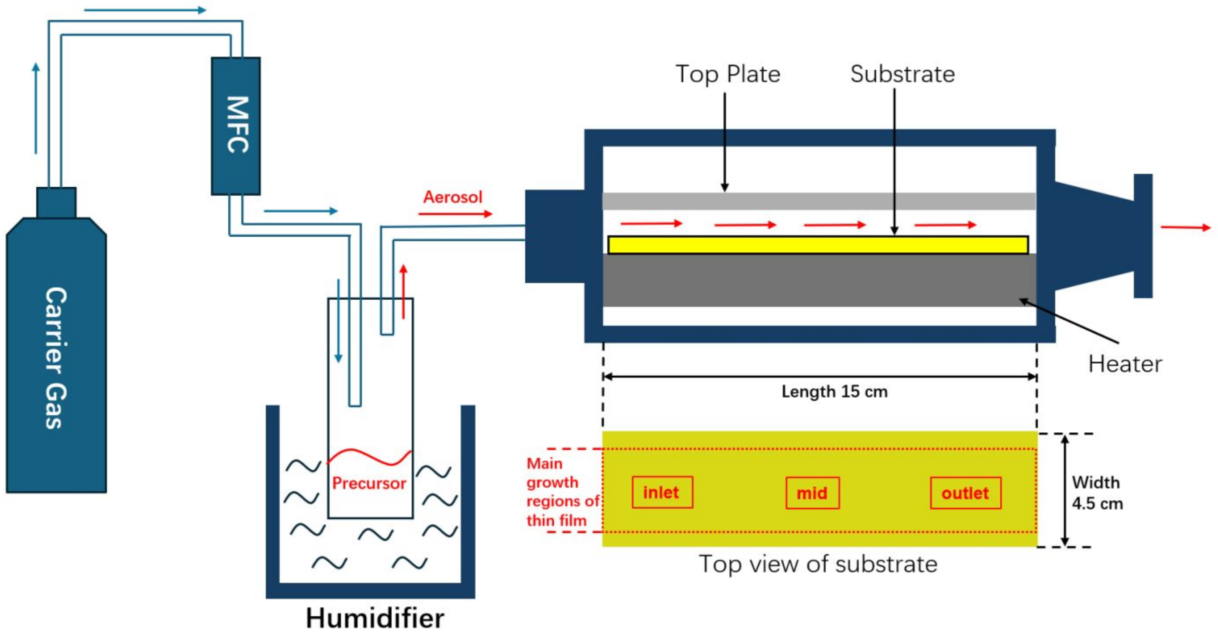

Figure S1. Schematic diagram of the aerosol-assisted chemical vapor deposition (AACVD) setup for thin film fabrication.

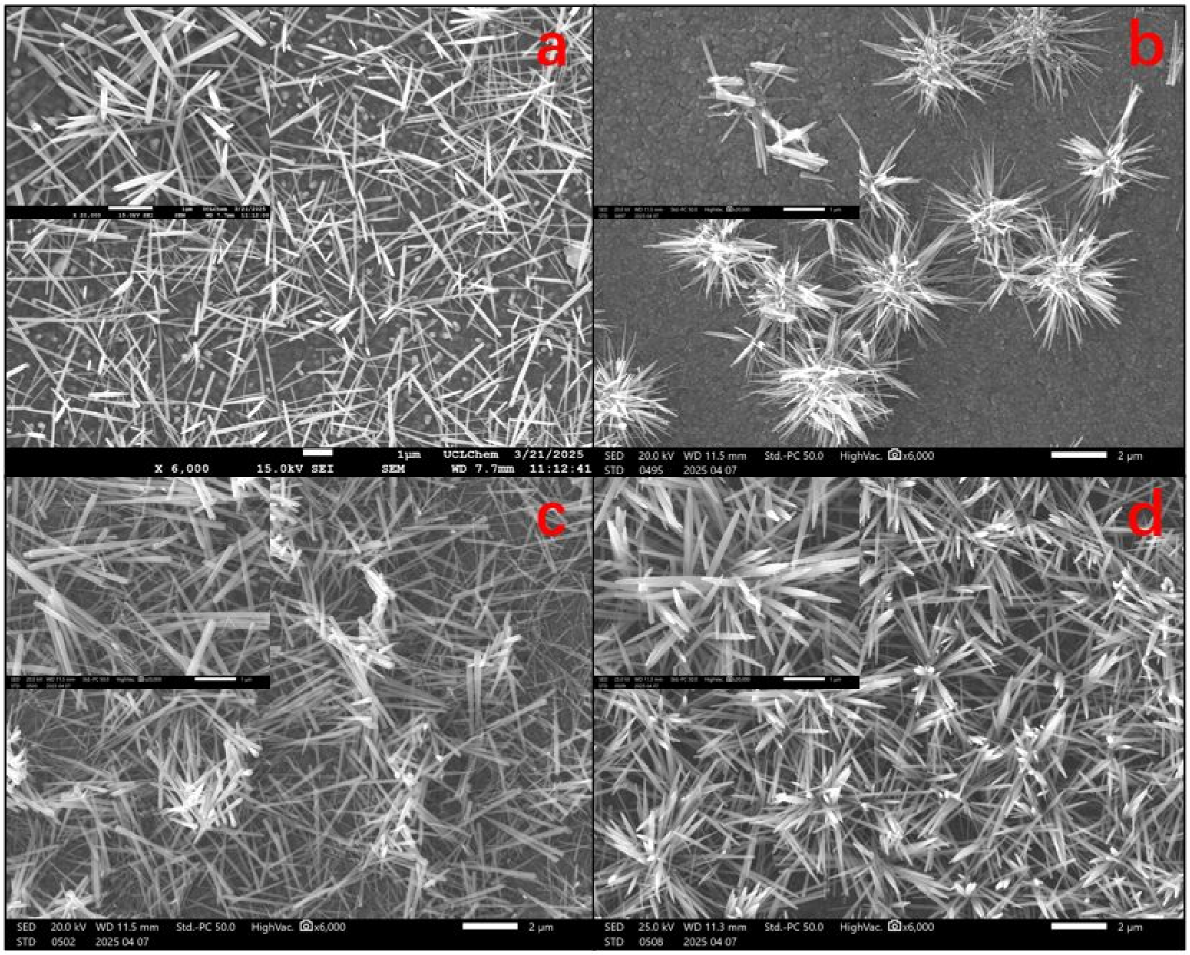

Figure S2. SEM comparison of $WO_3/BiVO_4$-mid samples synthesized with varying precursor volumes: (a) 20 mL, (b) 10 mL, (c) 30 mL, and (d) 40 mL.

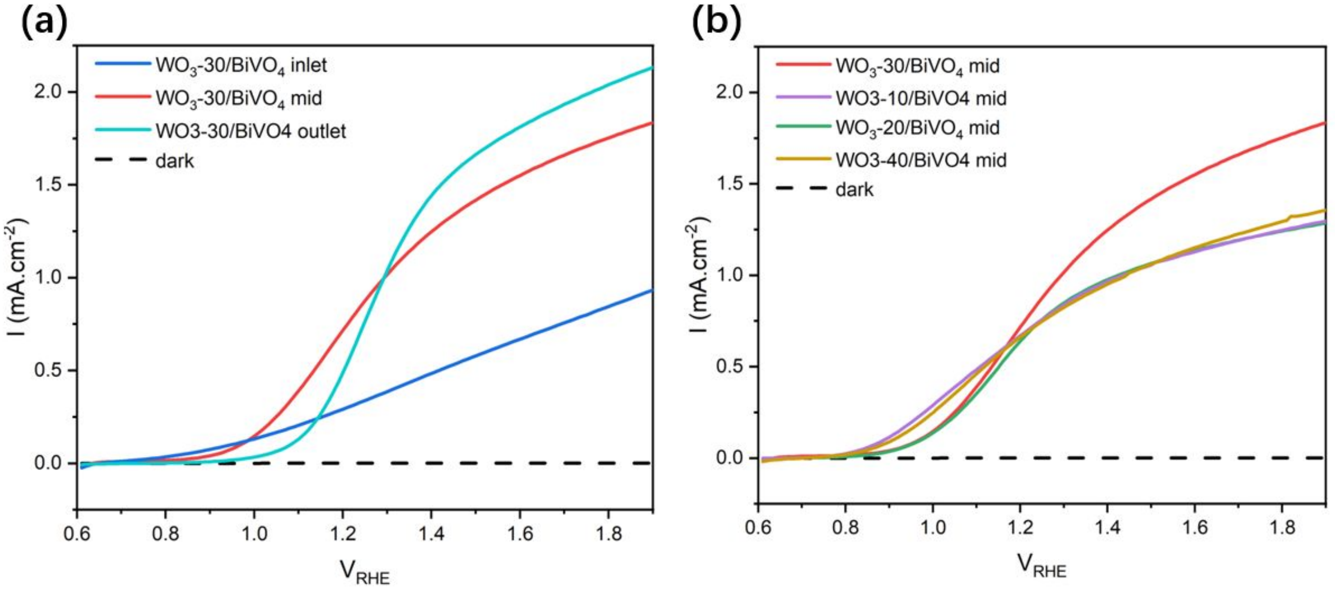

Figure S3. precursor (a) volume & (b) position-dependent PEC performance.

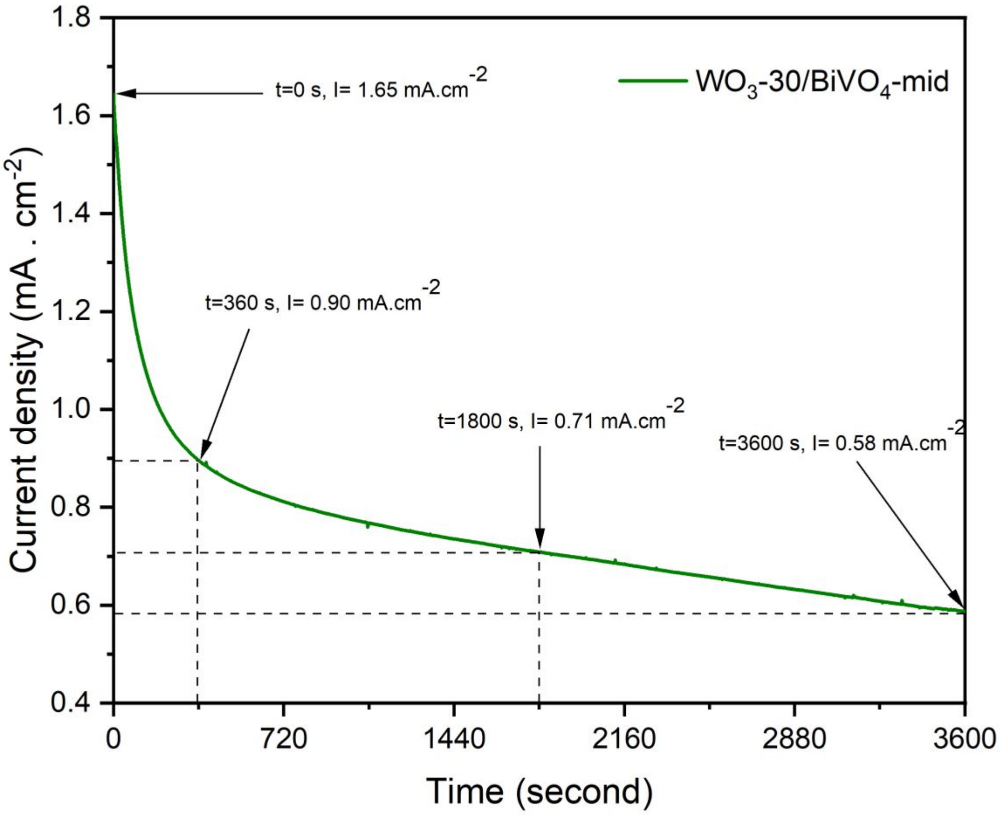

Figure S4. Chronoamperometry curve of the $WO_3/BiVO_4$ photoanode measured at 1.23 V_{RHE} under continuous illumination (AM 1.5 G).

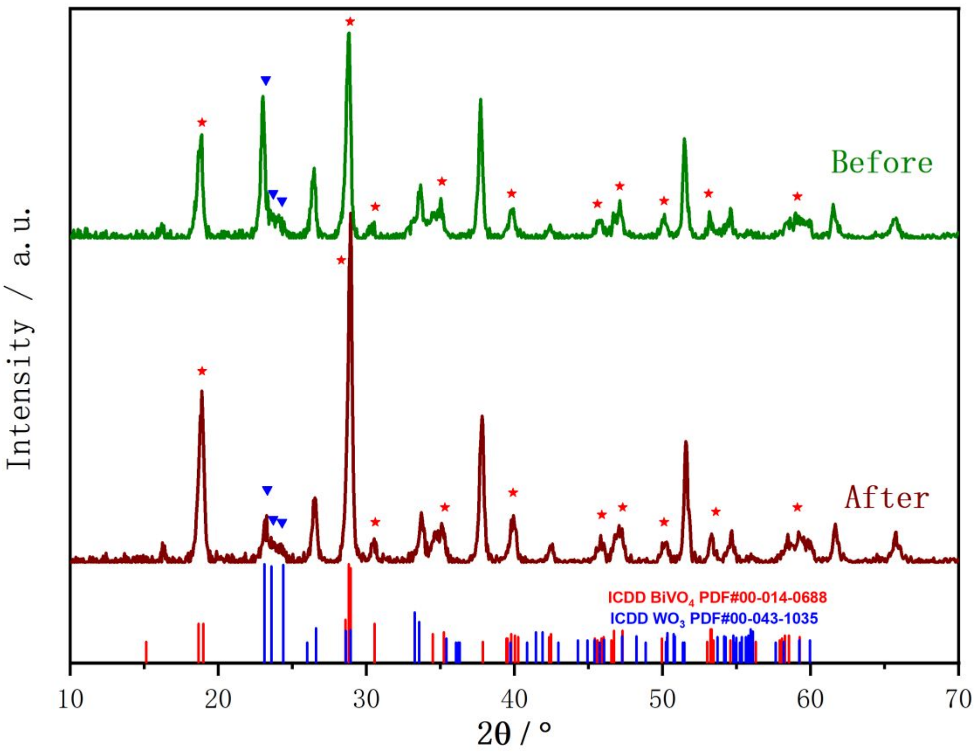

Figure S5. X-ray Diffraction patterns of the WO_3/$BiVO_4$photoanode before and after prolonged photoelectrochemical testing
